# Medicine Sellers for Prevention and Control of Sexually Transmitted Infections: Effect of a Quasi-Experimental Training Intervention in Bangladesh

**DOI:** 10.1155/2015/570340

**Published:** 2015-09-27

**Authors:** Nazmul Alam, Anadil Alam, Pierre Fournier

**Affiliations:** ^1^Global Health Unit, University of Montreal Hospital Research Centre (CR-CHUM), Montreal, QC, Canada H2X 0A9; ^2^School of Public Health, University of Montreal, Montreal, QC, Canada H3N 1X9; ^3^Centre for Reproductive Health, International Centre for Diarrhoeal Disease Research, Bangladesh (icddr,b), Dhaka 1212, Bangladesh

## Abstract

This study used a quasi-experimental pre-post design to test whether short training can improve medicine sellers' (MSs) practices and skills for prevention and control of sexually transmitted infections (STIs) in Bangladesh. The training included lectures, printed materials, and identification of referral sites. Difference-in-differences estimation was used to determine the effects of intervention on key primary and secondary outcomes. Advice given by the MSs in intervention group for partner treatment and condoms use increased significantly by 11% and 9%, respectively, after adjusting for baseline differences in education, religion, age, duration of training, and study site. Referral of clients to qualified service providers increased by 5% in the intervention group compared to the comparison group, but this change was not found to be statistically significant. Significantly higher proportion of MSs in the intervention group recognized the recommended medications as per the national syndromic management guidelines in Bangladesh for treatment of urethral discharge and genital ulcer symptoms. Short training intervention was found to be effective in improving MSs' practice of promoting condom use and partner treatment to the clients. We anticipate the need for broad based training programs of MSs to improve their skills for the prevention and control of STI/HIV in Bangladesh.

## 1. Introduction

Private-for-profit pharmacies areimportant sources of medicine, advice, and referrals for many people in low- and middle-income countries. People with symptoms of sexually transmitted infections (STIs) and other illnesses often seek health care in these pharmacies because they are affordable and easily accessible [1–3]. Most of these medicine shops do not have qualified pharmacists, however, and rather employ a cadre of “medicine sellers (MSs)” with or without professional training [4]. In some countries, the potential role of MSs in the prevention of STIs/HIV has been recognized and advocated at the policy level in order to formally acknowledge and effectively materialize their contribution to STI/HIV prevention and control. Some countries have adopted the training of MSs on syndromic management of STIs for the sake of providing quality services [5, 6]. These efforts have demonstrated positive impacts on the selection of proper drugs, counselling to use condoms, and referrals to qualified physicians [5, 7]. The World Health Organization (WHO) consultative group highlighted that the role of pharmacists has been changing over the past two decades with the increase of self-treatment worldwide [8].

Prevalence of STIs among the general population in Bangladesh is low but is high among the most-at-risk populations [9–12]. Prevalence of common STIs among women attending antenatal clinics was reported to be less than 5%, while prevalence of gonorrhoea and chlamydial infection was found to be 36% and 44%, respectively, among female sex workers in Bangladesh [10, 11]. Clinics run by Non-Government Organizations (NGOs) at the primary health care level, government hospital outpatient clinics, and practices at the private general practitioner's chamber play a vital role in managing STIs in Bangladesh, where syndromic management protocols are mostly used without the benefit of laboratory confirmations. Bangladesh adopted WHO recommended national STI syndromic management guidelines in 1999 and later updated them in 2006 [13, 14].

In Bangladesh, pharmacies sell medicine either based on doctors' prescriptions or on demand. There is no functioning prescription regulation on purchases of antibiotics. In addition to dispensing medicine, MSs also diagnose and treat illnesses, despite having no professional training. According to the Directorate of Drug Administration, there are approximately 70,000 registered drug retailers in Bangladesh and an additional 30,000 without licenses [15]. A study conducted among informal health providers in rural Bangladesh reported that only half of the MSs working in drug stores had any kind of training, leaving the other half without any kind of training whatsoever [16, 17]. Previous studies in Bangladesh and other developing countries reported that MSs had difficulties in recognizing STI symptoms, offered medications not recommended, seldom referred clients to qualified physicians, and were less likely to counsel patients for condom use or partner treatment [5, 7, 18, 19].

A number of studies, mostly cross-sectional in nature, have been conducted among MSs in Bangladesh, while most of them called for training programs to improve their skills for the prevention and control of STIs/HIV in the country [17, 19, 20]. This study aims to evaluate the role of short training intervention for MSs in pharmacies in and near selected brothels and truck-stand areas in Bangladesh in improving their knowledge and skills in recognizing STIs, preventing STIs/HIV, and providing referrals to formal providers for better health management.

## 2. Materials and Methods

This study was conducted between September 2009 and December 2010 using a quasi-experimental, pre-post design with an intervention and comparison group. Two brothel sites were purposively selected in Faridpur and Tangail districts, and two truck-stand sites were selected in Dhaka and Chittagong districts in Bangladesh. The two brothel sites were more or less similar in terms of number of sex workers they host and the number of pharmacies within one-kilometre radius of the brothels. Similarly, the two truck-stand sites were similar in terms of the number of trucks they hosted and the number of pharmacies within one-kilometre radius. Brothel and truck-stand sites were selected based on the assumption that care seeking for STI symptoms at the pharmacies would be higher in areas where vulnerable population for STIs is predominant. Sex workers are considered as high-risk groups for the acquisition and transmission of STIs/HIV, and truck drivers and their helpers are considered as potential clients of sex workers; there would presumably be a higher incidence of STI symptomatic individuals seeking care at pharmacies in their vicinity.

One brothel site and one truck-stand site were randomly selected as intervention areas, while the other two sites remained as comparison areas. Research assistants listed all private-for-profit pharmacies in the study areas who trade modern medicines and identified all MSs in each pharmacy along with their name, age, and duration of work. The MSs included in the study as study participants were of those who had been working in the targeted pharmacies for at least one month before the census was done. Pharmacies involved in selling homeopathic, traditional, and herbal medicines were not included in the study. The study protocol was approved by the ethical review committee of the International Centre for Diarrhoeal Diseases Research, Bangladesh (icddr,b), where this project was housed in Bangladesh. Written consent was taken from each participating subject during baseline and follow-up surveys. Intervention in this study included participation in training, distribution of printed materials, and establishment of local level referral linkages.

### 2.1. Training Workshop

Eligible MSs from pharmacies in the intervention areas were invited to participate in a two-day training session organized locally by the project. The MSs were grouped into 25–30 participants to allow more interactive sessions, while presentations and discussions were done in the local language. A team of three trainers conducted the training sessions, which included one medical doctor, one public health expert, and one health administrator with experience in treating STIs and in health systems in Bangladesh. The same team of trainers conducted the sessions in the intervention area. The training curriculum included an overview of human reproductive systems, the burden of STIs/HIV, aetiology and symptoms of common STIs, importance of patient referral, and prevention approaches. The first day of the training focused on human reproductive systems, aetiology and symptoms of common STIs, and the burden of STIs/HIV globally and in Bangladesh in particular. The second day of the training focused on the importance of patient referral, prevention approaches including condom use, and discussion on the national syndromic STI management guidelines to introduce suggested treatment options for major STI syndromes.

### 2.2. Distribution of Learning Materials

Each training participant received printed materials on STIs/HIV/AIDS, a copy of the national STI syndromic management guidelines, and a list of referral sites in their respective areas.

### 2.3. Establishing Referral Linkage

We prepared a list of possible referral linkages in the intervention sites and shared them with the MSs in the intervention group. Referral linkages included qualified general medical practitioners specializing in STIs, NGO clinics, and government hospitals offering treatment of sexually transmitted infections. We contacted each of the referral sites to discuss the project and encouraged them to accept patients referred from the participating MSs. Each participating MS in the intervention group received a list with addresses of such providers in their respective localities.

### 2.4. Data Collection

Data collection was done by the male interviewers at baseline and six months after the training using a semistructured questionnaire and data collectors were not blinded to the intervention. Data were collected by face-to-face interview of the MSs on the demographic characteristics, training, duration of work as a MS, knowledge about STI/HIV/AIDS, and practices with clients seeking care for STI symptoms in terms of referral, advice for condom use and partner treatment, and so forth. The questionnaire was pilot tested with MSs in other pharmacies outside the study area. The quality of data was maintained by field level scrutiny of each completed survey by the field supervisor for completeness and consistency. Study investigators made unscheduled visits to the study sites, reviewed completed questionnaires, and provided necessary advice to the interviewers. Collected data were entered in the Oracle database system with built-in error checking provisions by an experienced data entry operator after editing and coding the open ended responses.

### 2.5. Study Outcomes

Primary outcome variables included referral of patients with symptoms of STIs, advice to use condoms, and advice for partner treatment. Secondary outcome variables included knowledge of common STIs, mode of STI transmission, knowledge about drug resistance, knowledge about national STI syndromic guidelines, and recommendation of drugs for treatment of genital ulcer symptoms, urethral discharge, and abnormal vaginal discharge.

Sample size was calculated to be 220 MSs (110 each in the intervention and comparison group) to detect a difference of 25% in key outcome variables between the intervention and comparison groups, with 80% power (Type II error) and a 5% significance level (Type I error). This 25% difference refers to 50% of cases with symptoms of STIs referred by the informal service providers to qualified providers in Bangladesh [20], to be increased to 75% after intervention.

### 2.6. Data Analyses

Descriptive analyses were performed to summarize and compare the demographic characteristics (age, education, training level, etc.) of the MSs in the intervention and comparison groups. Chi-squared tests were used to compare categorical values, and independent sample *t*-tests were used to compare continuous variables. Key variables from each of these categories were compared between the intervention and comparison groups both at the baseline and the follow-up. Difference-in-differences (DiD) estimation was used to assess the effects of training on key outcome variables in the framework of a linear regression model [13, 21]. We defined *i* = 1 for the intervention group and *i* = 0 for the control group and *t* = 1 as the end line and *t* = 0 as the baseline. The DiD estimator is the coefficient of the interaction term between intervention and time in a linear regression model with intervention, time, and their interaction as covariates. For binary outcome variables, the DiD estimate is the difference from the baseline to the end line in terms of proportion of MSs reporting an event in the intervention and control group. The difference in proportion between the intervention and control group at the end line is the unadjusted estimate of the intervention effect. In the adjusted model, education, religion, type of study sites (truck stand and brothel site), and age and training of the MSs were included as covariates based on baseline differences observed with these variables. Differences in proportions observed in secondary outcomes were calculated from baseline to end line separately for the comparison and intervention groups, and net DiD were estimated along with their corresponding *p* values using Chi-squared tests. Data analysis was done using SPSS 20 and STATA 11.

## 3. Results

A total of 269 MSs participated in the baseline survey, 118 in the comparison group (CG) and 151 in the intervention group (IG); this represents 89% of the 133 MSs originally identified in the census in the CG from 46 pharmacies and 91% of the 166 identified in the IG from 55 pharmacies. In the end line survey, 116 MSs participated in the CG and 138 in the IG, representing 87% and 83% of those identified in the CG and IG, respectively. There were, approximately, three MSs per pharmacy enlisted in both the CG and IG. Approximately half of the MSs were recruited from truck-stand areas and another half from brothel areas (Table 1). MS was found to be predominantly a male profession (>99%) in Bangladesh, and the highest proportion of the MSs belonged to the age group of 26–35 years ranging from 38% to 43%.

### 3.1. Formal Training

At baseline, all of the MSs had at least some level of education, while 42% and 30% of the MSs had more than twelve years of education in the CG and IG, respectively. Type of professional training of the MSs at baseline ranged from no training (29% in CG and 40% in IG) to paramedical training (5% in CG and 2% in IG), local medical assistant (LMA) training (49% among CG and 41% among IG), and pharmacist training (33% among CG and 50% among IG). Nearly a quarter of the MSs had been involved for 5 years or less in this profession, while another quarter of them have been working for more than 15 years.

### 3.2. Practices

There were wide variations in the number of clients with STI symptoms who sought care to the MSs, representing 0 to 30 in the CG and 0 to 85 in the IG at baseline and 0 to 60 in CG and 0 to 38 in the IG at end line (Table 2). For both male and female clients, MSs predominantly depended only on the history of the clients for recognising the STI status, and only a small proportion of them used a combination of history taking, physical examination, and laboratory investigations in both the CG and IG at both the baseline and end line surveys.

Only a third of the MSs reported having a private space to take history of the clients and do physical examination to the clients as reported during the baseline survey; by the end line survey it increased in both the CG (43%) and IG (48%). All of the MSs reported selling medicine to their clients, but only a small proportion received fees for their consultation services in both the CG and IG.

### 3.3. Effect of Training on Primary Outcomes of Prevention and Referral Practices

In the IG, 11% of the MSs advised their clients for partner treatment, 68% advised to use condoms, and 57% referred their clients at baseline and those proportions increased to 29%, 82%, and 69%, respectively, at end line. In the CG, 12% of the MSs advised their clients for partner treatment, 69% advised to use condoms, and 61% referred their clients at baseline and those proportions increased to 17%, 73%, and 67%, respectively, at end line.  Table 3 presents the effects of intervention from DiD estimations on the three main outcome variables. There was a significant increase in the practice of MSs to advise clients for partner treatment in the IG compared to the CG from baseline to the end line by 11% after adjustment of baseline differences of education, religion, study sites, age, and types of training. Likewise, there was a significant increase in the MSs' practice of advising their clients to use condoms, 9% in the IG compared to the CG. Although MSs' practice of referring their clients with STI symptoms to qualified providers increased by 5% in the IG, this change was not found to be statistically significant.

### 3.4. Effect of Training on Secondary Outcomes of Knowledge and Use of Medication for STI Management

Knowledge about HIV and other common STIs was found to be high during the baseline survey, ranging from 88% to 100% in both CG and IG (Table 4). By the end line survey, knowledge about HIV, gonorrhoea, syphilis, and abnormal vaginal discharge was found to be 100%, 99%, 97%, and 98% in the CG and 100%, 99%, 98%, and 99% in IG, respectively. There was no significant change in the proportion of MSs who answered correctly three or four methods of HIV transmission (unprotected sexual intercourse, blood transfusion, needle sharing, and infected mother to child). Net difference in change of knowledge about availability of national STI syndrome management guidelines was found to be significantly higher in IG during the end line survey. Knowledge of antibiotic abuse contributing to drug resistance increased significantly in the IG, from 40% to 60% after the training. A positive change was observed in the CG, from 37% to 52%; however, this net difference was not found to be statistically significant.

**Figure 1:**
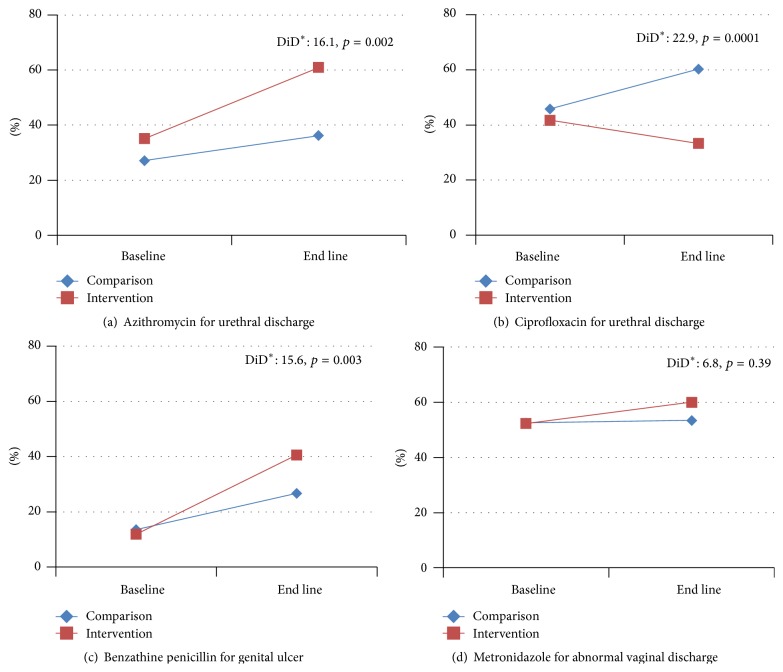
Proportion of medicine sellers who recognized antibiotics as per national syndromic management guideline for selected symptoms of sexually transmitted infections. DiD^*∗*^: difference in differences.

The most significant changes in the recommendation of azithromycin for treatment of urethral discharge (gonorrhoea) and benzathine penicillin for treatment of genital ulcer were observed in the IG as per the national syndromic management guidelines (Figure 1). At the baseline, 35% of the MSs recommended azithromycin for urethral discharge; this number increased to 61% by the end line survey (*p* = 0.002), which represents a 16% absolute change in the IG compared to the CG. Recommendation of benzathine penicillin for the management of syphilis showed a 16% absolute increase in the IG compared to the CG. Recommendation of metronidazole for management of abnormal vaginal discharge also changed after the intervention, 52% at baseline to 60% at the end line in the IG (*p* = 0.39). Ciprofloxacin is not a drug of choice by the national syndromic management guidelines because of high levels of resistance for* Neisseria gonorrhoeae*, a causative agent of gonorrhoea. Absolute decrease in the choice of ciprofloxacin for the treatment of urethral discharge was observed to be 23% after the training (*p* = 0.001).

## 4. Discussion

The results of this study showed that a short training session of two days improved MSs' reported practices in rendering prevention services for their clients seeking care for STI symptoms. A significantly higher proportion of the MSs in the IG reported to advise their clients for partner treatment and use of condoms. MSs are well positioned to contribute to the STI/HIV prevention campaign through their client base and wide accessibility to the general population in many low- and middle-income countries [5, 7, 22, 23]. One of the key objectives of this study was to increase the referral of clients with STI symptoms to qualified providers. Only a moderate increase in referrals was observed in the IG after adjustment of the difference observed in the CG, which may be explained by the fact that MSs do not anticipate any financial incentives by referring a client, since they cannot sell medicine to them. It could be plausible that they became more confident to handle cases with STIs after participation in the training sessions. Higher proportion of MSs advised their clients for partner treatment and for condom use may have links with their business pursuit as well but these practices are recognized STI/HIV prevention intervention.

All of the MSs in this study sell medicine to their clients as principal source of income, while most of them do not take fees for consultation. This creates a powerful incentive to overmedicate clients seeking care from them. This finding was also suggested by other studies in Bangladesh and elsewhere [6, 18]. MSs solely depend on knowing histories of the clients in their assessment of diagnosing STIs, a practice which leaves sufficient room for misdiagnosis and offering inappropriate medications as treatment. Less than half of the MSs relied on physical examination and laboratory investigations for further evaluation of STIs; we, however, did not investigate the quality and type of drugs or the type of laboratory investigations that were recommended. Medicine sellers in this study had inadequate training and in fact, they are not mandated from regulatory point of view in Bangladesh to advise medications especially any antibiotics to their clients.

Bangladesh adopted a set of national STI syndromic management guidelines in 1999 and later updated them in 2006, but less than a quarter of the MSs knew about these at the baseline. However, knowledge of MSs about the guidelines and the recommended medication for common STIs significantly increased in the IG, especially for urethral discharge syndrome and genital ulcer diseases. Positive effects of intervention were further supported by the fact that nearly half of the MSs in the IG dropped their recommendation of ciprofloxacin for urethral discharge symptoms (gonorrhoea), a drug which is not a choice in the national guidelines because of high levels of ciprofloxacin-resistant gonococci strains in Bangladesh [3, 24]. There is still considerable room for improvement in MSs' knowledge, particularly in how STI/HIV transmission occurs and knowledge of antibiotic abuse and the development of drug resistance; perhaps a longer training program would have a greater impact. Study conducted among pharmacy workers [9, 17, 25, 26] and unqualified “village doctors” [20] highlighted the importance of providing training to MSs to improve their skills considering their role as informal care providers in low- and middle-income countries. Our study reinforces the importance of such training targeting medical sellers and other informal providers in Bangladesh context to strengthen STI/HIV prevention campaign.

Major strength of this study was having a comparison group in the design to enable assessment of influences beyond the intervention. Moreover, we used difference-in-differences measures to account for the net changes observed in the intervention group and to take care of the changes observed in the comparison group. This study had some limitations which need to be pointed out. First, it was conducted in four purposively selected geographic locations, which had a small study size. This limits the potential to achieve statistical significance of differences in some cases. We have collected self-reported data from the MSs, which may have suffered from social desirability bias for the outcome variables in the intervention group; hence, caution is needed to interpret and generalize the study's findings. Second, we cannot completely rule out the possibility of information sharing having occurred, as the MSs in the two groups may have had the opportunity to meet. However, the intervention and comparison areas chosen were geographically isolated, limiting the chance of contamination of information. Third, it would have been ideal to conduct analysis using the clusters (study sites) as the unit of analysis, considering the design of the study, but we considered only study subjects as the unit of analysis, which can spuriously overestimate the significance of differences. It was not feasible to consider study sites as the unit of analysis because we had only two intervention sites and two comparison sites with small sample sizes; instead, we have adjusted the DiD estimates for study sites along with other covariates.

It is evident from the findings of this and related studies [18–20] that MSs are capable of improving their skills through short training sessions to contribute to the prevention of STIs in Bangladesh. Syndromic management approach for STIs was found to have limitations among women in Bangladesh because women might have infections without any symptoms but the approach has been advocated for men [9]. We call for further operations research to assess the feasibility of involving MSs for STI syndromic management especially among men in Bangladesh context.

## 5. Conclusion

Short training to MSs in private-for-profit pharmacies in Bangladesh contributed to increase their practices to advise the clients with STI symptoms to use condoms and seek partner treatment. We anticipate the need for nationwide training of MSs to improve their skills to make significant strides in STI/HIV prevention campaigns in Bangladesh.

## Figures and Tables

**Table 1:** Sociodemographic characteristics of the medicine sellers at baseline and end line study by intervention and control group.

Characteristics	Baseline		End line	
*N* (%)	Comparison (*n* = 118)	Intervention (*n* = 151)	Comparison (*n* = 116)	Intervention (*n* = 138)
Study sites				
Truck stand	58 (49.2)	90 (59.6)	55 (47.4)	90 (65.2)
Brothel	60 (50.8)	61 (40.4)	61 (52.6)	48 (34.8)
Age				
16–25 years	16 (13.6)	28 (18.5)	15 (12.9)	19 (13.8)
26–35 years	47 (39.8)	63 (41.7)	44 (37.9)	55 (39.9)
36–45 years	26 (22.0)	38 (25.2)	27 (23.3)	43 (31.2)
≥46 years	29 (24.6)	22 (14.6)	30 (25.9)	21 (15.2)
Sex				
Male	118 (100)	150 (99.3)	116 (100)	137 (99.3)
Female	0 (0.0)	1 (0.7)	0 (0.0)	1 (0.7)
Education				
5–10 years	25 (21.2)	48 (31.8)	29 (25.0)	25 (18.1)
11-12 years	43 (35.4)	57 (37.7)	43 (37.1)	68 (49.3)
13–18 years	50 (42.4)	46 (30.5)	44 (37.9)	45 (32.6)
Religion				
Muslim	97 (82.2)	72 (47.7)	95 (81.9)	69 (50.0)
Others	21 (17.8)	79 (52.3)	21 (18.1)	69 (50.0)
Duration of training				
No formal training	34 (28.8)	61 (40.4)	29 (25.0)	39 (28.2)
<6 months of training	14 (11.9)	17 (11.3)	23 (19.8)	33 (23.9)
6–12 months of training	56 (47.5)	58 (38.4)	50 (43.1)	55 (39.9)
>1 year of training	14 (11.8)	15 (9.9)	14 (12.1)	11 (8.0)
Type of training received	*n* = 84	*n* = 90	*n* = 87	*n* = 99
Paramedical	4 (4.8)	2 (2.2)	1 (1.1)	3 (3.0)
LMAF	41 (48.8)	37 (41.1)	34 (39.1)	44 (44.5)
Pharmacist	28 (33.3)	45 (50.0)	47 (54.0)	48 (48.5)
Others	11 (913.1)	6 (6.7)	5 (5.7)	4 (4.0)
Duration of work as a medicine seller				
≤5 years	30 (25.4)	46 (30.5)	28 (24.1)	31 (22.5)
6–10 years	41 (34.7)	38 (25.2)	41 (35.3)	42 (30.4)
11–15 years	18 (15.3)	30 (19.9)	16 (13.8)	31 (22.5)
≥16 years	29 (24.6)	37 (24.5)	31 (26.7)	34 (24.6)

**Table 2:** Practices of medicine sellers to the clients with symptoms suggesting sexually transmitted infections.

Characteristics	Baseline		End line	
*N* (%) unless otherwise specified	Comparison	Intervention	Comparison	Intervention
	(*n* = 118)	(*n* = 151)	(*n* = 116)	(*n* = 138)
Patients with STI symptoms seen in the last month (median, range)				
Male patients	4 (0–30)	5.0 (0–85)	6 (0–60)	7 (0–38)
Female patients	2.5 (0–30)	3 (0–50)	3.0 (0–25)	4 (0–22)
Method of STIs assessment in men^*∗*^				
History only	82 (69.5)	132 (87.4)	68 (58.6)	85 (61.6)
History and physical exam.	34 (28.8)	15 (9.9)	46 (39.7)	49 (35.5)
History and laboratory investigations	7 (5.9)	6 (4.0)	11 (9.5)	6 (4.3)
Method of STIs assessment in women^*∗*^				
History only	88 (74.6)	110 (72.9)	110 (94.8)	123 (89.1)
History and physical exam	3 (2.5)	0	11 (9.5)	8 (5.8)
History and laboratory investigations	30 (25.4)	41 (27.1)	5 (4.3)	15 (10.9)
Given paper prescription to patients				
Always	39 (33.1)	23 (15.2)	58 (50.0)	58 (42.0)
Sometimes	69 (58.5)	108 (71.6)	53 (45.7)	77 (55.8)
Never	10 (8.4)	20 (13.2)	5 (4.3)	3 (2.2)
Got private space for physical examination	43 (36.4)	47 (31.1)	50 (43.1)	66 (47.8)
Received consultation fee from patients	1 (0.8)	13 (8.6)	2 (1.7)	12 (8.7)

^*∗*^Multiple responses accepted.

**Table 3:** Effect of training on three major outcome variables based on difference-in-differences (DID) estimation.

Outcome variable	Without covariate			With covariates^*∗∗*^		
	DID estimator^*∗*^ (SE)	*R* ^2^	*p* value	DID estimator (SE)	*R* ^2^	*p* value
Referred patients with STI symptoms in the last one month	0.114 (0.082)	0.165	0.116	0.052 (0.061)	0.132	0.369
Advised patients with STI symptoms to use condoms	0.121 (0.041)	0.131	0.003	0.093 (0.027)	0.110	0.04
Advised patients with STI symptoms for partner treatment	0.259 (0.035)	0.313	0.001	0.191 (0.041)	0.259	0.009

^*∗*^The difference-in-differences estimator was calculated using linear regression model, ^*∗∗*^adjusted for education, religion and sites (brothel or truck stand), age, and training of the MSs.

**Table 4:** Effect of training on knowledge of medicine sellers showing difference in differences.

Characteristics	Comparison			Intervention			DiD^*∗*^	*p* value
	Baseline	End line	Difference	Baseline	End line	Difference		
	(*n* = 118)	(*n* = 116)		(*n* = 151)	(*n* = 138)			
Knowledge about								
HIV	118 (100)	116 (100)	0	151 (100)	138 (100)	0		NS^*∗∗*^
Gonorrhea	107 (90.7)	115 (99.1)	8.4	136 (90.1)	137 (99.3)	9.2	0.8	NS
Syphilis	104 (88.1)	113 (97.4)	9.3	125 (82.8)	136 (98.6)	15.8	6.5	NS
Bacterial vaginosis	112 (94.9)	114 (98.3)	3.4	142 (94.0)	137 (99.3)	5.3	1.9	NS
Naming three or more correct methods of STI/HIV transmission	88 (74.6)	74 (63.8)	−10.8	101 (66.9)	94 (68.1)	1.2	12.0	NS
Knowledge of STI syndromic guideline	23 (19.5)	26 (22.4)	2.9	29 (19.2)	117 (85.0)	65.8	62.9	0.0001
Knowledge of drug resistance and antibiotic abuse	42 (36.8)	53 (51.5)	14.7	58 (39.5)	83 (60.3)	20.8	6.1	NS

^*∗*^DiD: difference in differences; ^*∗∗*^NS: not significant.
